# Development and validation of a nomogram for predicting recurrence in patients with Meige syndrome after radiofrequency ablation

**DOI:** 10.3389/fneur.2026.1841516

**Published:** 2026-07-14

**Authors:** Xiang Xu, Tao Wang, Zi Wang

**Affiliations:** 1 Department of Anesthesiology and Pain Medicine, Huzhou Wuxing District People's Hospital, Huzhou Wuxing District Maternal and Child Health Hospital, Huzhou, China; 2 Department of Anesthesiology and Pain Research Center, The Affiliated Hospital of Jiaxing University, Jiaxing, China

**Keywords:** Meige syndrome, nomogram, prediction model, radiofrequency ablation, risk factors

## Abstract

**Introduction:**

Radiofrequency ablation targeting the extracranial facial nerve has emerged as an effective therapeutic option for patients with Meige syndrome (MS) presenting predominantly with blepharospasm. However, postoperative recurrence remains a major clinical challenge. This study aimed to develop and internally validate a nomogram for predicting 1-year recurrence after CT-guided facial nerve radiofrequency ablation via bilateral stylomastoid foramen.

**Methods:**

This study retrospectively analyzed 119 patients with MS who underwent CT-guided facial nerve radiofrequency ablation via bilateral stylomastoid foramen at the Pain Department of Jiaxing University Affiliated Hospital between November 2021 and December 2024. The Lasso regression and multivariate logistic regression analysis were employed to identify independent factors associated with postoperative recurrence. A nomogram was constructed based on these factors and internally validated using the bootstrap resampling method.

**Results:**

Our analysis indicates that independent factors influencing recurrence are the Jankovic Rating score (JRS),disease duration, and final temperature. A nomogram was constructed based on these predictors. The area under the ROC curve (AUC) for the nomogram was 0.932 (95% CI: 0.912–0.958). Calibration curves demonstrated excellent agreement between predicted probabilities and actual observed values. Decision curve analysis (DCA) indicated that the nomogram predictive model had a higher net benefit for predicting the risk of postoperative recurrence in MS patients.

**Conclusion:**

This study constructed a static and dynamic online nomogram with excellent differentiation, calibration, and accuracy, aiding in the identification of high-risk postoperative patients and assisting physicians in decision-making.

## Introduction

Meige syndrome (MS) is a rare form of cranial dystonia primarily characterized by blepharospasm and oromandibular dystonia. Based on clinical manifestations, it can be classified into three subtypes: blepharospasm, blepharospasm–oromandibular dystonia, and oromandibular dystonia (1). In general, MS most commonly affects individuals aged 40 to 70 years, with a higher prevalence in females than in males (2). The etiology and pathophysiological mechanisms of MS remain unclear. Current evidence suggests that it is mainly associated with an imbalance in acetylcholine and dopamine neurotransmission, while environmental triggers and genetic susceptibility may induce neural plastic changes and reduced cortical inhibition (3, 4). Blepharospasm is the most common and disabling clinical manifestation in patients with MS, primarily involving involuntary contractions or tonic spasms of the orbicularis oculi muscle. Clinically, it often presents as excessive blinking (5). Approximately 25% of patients initially present with unilateral symptoms, which rapidly progress to bilateral involvement and subsequently spread to the lower face, mouth, jaw, tongue, and pharyngeal muscles; in rare cases, the condition may extend to the trunk and limbs (1, 6). Although MS is rare, it can severely impair daily functioning and social activities, and may even lead to psychiatric comorbidities such as anxiety and depression in some patients (7, 8).

Current treatment options for MS include oral medications, botulinum toxin injections, and surgical interventions (1). Oral medications generally provide limited clinical benefit, while the therapeutic effects of botulinum toxin type A injections are temporary and require repeated administrations to maintain efficacy (9–11). For patients who respond poorly to botulinum toxin type A, deep brain stimulation (DBS) targeting either the globus pallidus internus (GPi) or the subthalamic nucleus (STN) has become an established surgical treatment. Recent randomized controlled evidence demonstrated comparable efficacy between GPi-DBS and STN-DBS in improving motor symptoms and quality of life (12). Nevertheless, the high cost of surgery and associated materials often limits its accessibility (13, 14). Periocular surgery can alleviate blepharospasm symptoms in most patients, but it may also lead to complications such as dry eye and corneal exposure irritation, and in a minority of cases, postoperative worsening of blepharospasm may occur (15). In 2022, Huang et al. reported that CT-guided radiofrequency ablation of the bilateral facial nerve at the stylomastoid foramen can effectively treat patients with MS presenting predominantly with blepharospasm, with relatively low procedural cost and adverse events limited to class II–III facial paralysis (16). However, due to the small sample size and the lack of studies investigating postoperative recurrence, further evidence is needed. Therefore, the present study retrospectively analyzed the clinical data of patients with MS, explored the risk factors for recurrence, and developed a predictive model, aiming to provide a reference for preventing recurrence following radiofrequency ablation.

## Methods

### Study design and study population

**Figure 1:**
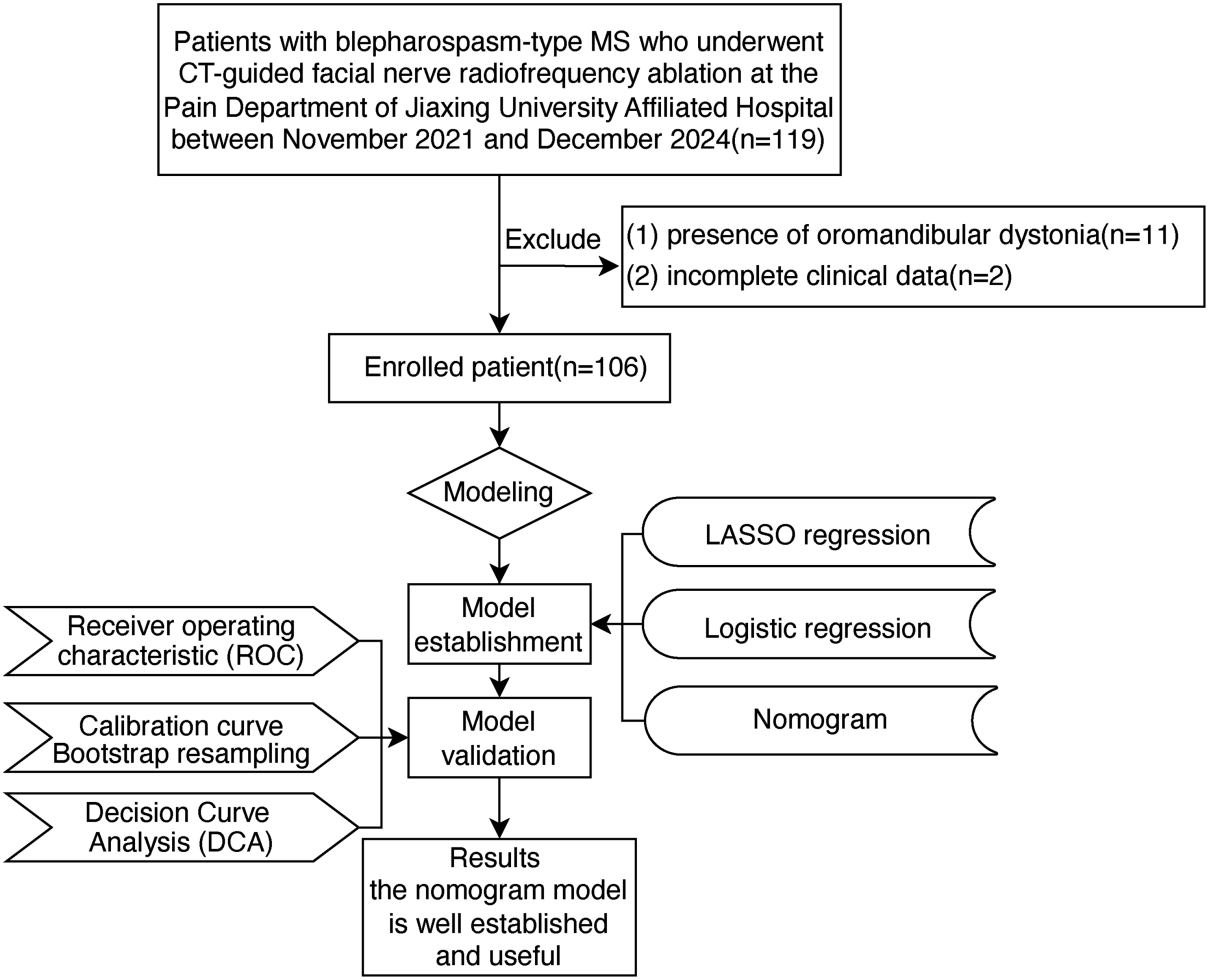
The overall flowchart of the study.

This study received approval from the Ethics Committee of the Affiliated Hospital of Jiaxing University (2026LP076) and was conducted by the Declaration of Helsinki and clinical practice guidelines. The study retrospectively gathered data from 119 MS patients who underwent CT-guided radiofrequency ablation of bilateral stylomastoid foramen facial nerve in the Pain Department of the Affiliated Hospital of Jiaxing University between November 2021 and December 2024. Inclusion criteria were as follows: (1) meeting the diagnostic criteria for MS (1); (2) Jankovic Rating Scale (JRS) score (17) > 5; (3) refractory to or intolerant of first-line medical therapies, including oral medications and/or botulinum toxin injections; (4) refusal of DBS therapy. Exclusion criteria included: (1) presence of oromandibular dystonia or blepharospasm–oromandibular dystonia; (2) a history of Parkinson’s disease or tremor, long-term use of psychosedative medications, craniocerebral trauma, cerebrovascular accident, or encephalitis; (3) Previous DBS therapy; (4) contraindications to radiofrequency ablation, such as coagulation disorders, infection at the puncture site, or the presence of a cardiac pacemaker; (5) incomplete clinical data. The restriction of enrollment to MS was based on the specific treatment target of this study. CT-guided radiofrequency ablation via the stylomastoid foramen targets the extracranial facial nerve, which is the principal motor pathway driving orbicularis oculi muscle contractions. In contrast, oromandibular dystonia or blepharospasm–oromandibular dystonia involve additional cranial nerves, such as the trigeminal and hypoglossal nerve systems, which cannot be anatomically addressed by facial nerve ablation. All consecutive eligible patients who met the inclusion and exclusion criteria during the study period were included, resulting in a final study cohort of 106 patients. The study flowchart is presented in Figure 1.

### Procedural methods

Patients were placed in the lateral decubitus position on the CT table, with continuous monitoring of heart rate, blood pressure, and oxygen saturation. A CT positioning grid was placed at the earlobe, and a paranasal sinus CT scanning protocol was used to perform 3-mm slice scans of the mastoid region. The stylomastoid foramen was identified and designated as the puncture target. The CT slice demonstrating the stylomastoid foramen without bony obstruction from the tympanic part of the temporal bone was selected as the puncture plane. Using CT measurement software, a straight line was drawn anteriorly from the stylomastoid foramen, and the intersection of this line with the skin surface was defined as the puncture point. After routine skin disinfection and local anesthesia, a 7-gauge radiofrequency needle (model 240,100; Innomed Medical Technology Co., Ltd.) with a length of 10 cm and a 5-mm active tip was advanced stepwise toward the target under CT guidance, and correct positioning was confirmed by CT scanning. Subsequently, motor nerve stimulation was performed using low-frequency (2 Hz) electrical current. Facial muscle contractions at the same frequency as the stimulation could be elicited at a current of 0.5 mA. During radiofrequency ablation, patients were instructed to inflate the cheeks and close their eyes tightly. The initial ablation temperature was set at 60 °C for 30 s. During the procedure, careful observation was required to assess whether the patient could maintain cheek puffing and tight eye closure on the treated side, in order to detect air leakage. If the patient exhibited difficulty in closing the eyes or air leakage occurred, ablation was immediately terminated and the procedure concluded. After 30 s of ablation, if no air leakage was observed during cheek puffing and forceful eye closure, the temperature was increased by 5 °C in the subsequent cycle. The temperature was incrementally increased in 5 °C steps until air leakage occurred during cheek puffing on the treated side and the patient was unable to maintain tight eye closure. The same procedure was then performed on the contralateral side[16].

### Data collection

Clinical data were collected using the Haite Electronic Medical Record System (version 3.0), including sex, age, disease duration, Jankovic Rating Scale (JRS) score, comorbidities (hypertension and anxiety), history of botulinum toxin treatment, operative time, and termination temperature. The JRS score at 1 year postoperatively was obtained through outpatient visits or telephone follow-up. The primary outcome was recurrence, defined as (preoperative JRS − postoperative JRS)/preoperative JRS < 50% (18).

### Statistical analysis

Statistical analyses were performed using R software (version 4.3.1). Continuous variables with a normal distribution were expressed as mean ± standard deviation, whereas non-normally distributed data were presented as median (interquartile range, IQR). Categorical variables were reported as counts (percentages). Group comparisons for continuous variables were conducted using the independent samples t-test or the Mann–Whitney U test, as appropriate, while categorical variables were analyzed using the chi-square test. The R packages “comparegroups,” “glmnet,” “glm,” “regplot,” “ggplot,” “ggROC,” “DynNom,” “rms,” “rmda,” “ROCR,” “rsconnect,” and “Dcurves” were used for data processing and visualization. Feature selection is a critical step in model development. In this study, the least absolute shrinkage and selection operator (LASSO) algorithm was applied to identify relevant features (19, 20). Subsequently, multivariable logistic regression analysis was performed based on the features selected by LASSO to determine statistically significant predictors. Variance inflation factors (VIFs) were calculated to mitigate the potential impact of multicollinearity (21).

A nomogram was then constructed based on the selected predictors. The discriminative ability of the predictive model was quantified using the area under the receiver operating characteristic curve (AUC), with values approaching 1.0 indicating excellent predictive performance (22). Model calibration was assessed using calibration plots, with internal validation performed via 500 bootstrap resamples to reduce overfitting (23). To evaluate clinical utility, decision curve analysis (DCA) was conducted to estimate the net benefit across a range of threshold probabilities. In addition, clinical impact curves (CIC) and net reduction in interventions were analyzed to estimate the potential clinical consequences at the population level (24).

## Results

### General information

A total of 106 patients were included in the final analysis, all patients completed the 1-year follow-up, comprising 70 males (66%) and 36 females (34%), with a mean age of 59.02 ± 8.98 years. All patients achieved significant symptom relief on the first postoperative day, with JRS scores ≤ 2 in all cases. No instances of primary treatment failure were observed in this cohort. At the 1-year follow-up, 35 patients experienced recurrence, yielding a recurrence rate of 33%. Univariate analysis of the selected variables (Table 1) demonstrated that age, disease duration, JRS score, history of botulinum toxin (BTX) treatment, and final ablation temperature were significantly associated with postoperative recurrence (*p* < 0.05). In contrast, the remaining variables were not statistically significant (*p* > 0.05).

**Table 1:** Comparison of general information between the non-recurrence and recurrence patients.

Variables	Total (*n* = 106)	Non-recurrence (*n* = 71)	Recurrence (*n* = 35)	*p* value
Gender, *n* (%)				0.866
Male	70 (66)	46 (65)	24 (69)	
Female	36 (34)	25 (35)	11 (31)	
Age (years), mean ± SD	59.02 ± 8.98	57.59 ± 9.27	61.91 ± 7.68	0.013
Duration (years), Median (Q1–Q3)	3 (2–6)	2 (1–3.5)	8 (5–10)	<0.001
JRS, *n* (%)				<0.001
6	67 (63)	63 (89)	4 (11)	
7	23 (22)	8 (11)	15 (43)	
8	16 (15)	0 (0)	16 (46)	
BTX history, *n* (%)				0.037
No	62 (58)	47 (66)	15 (43)	
Yes	44 (42)	24 (34)	20 (57)	
Hypertension, *n* (%)				0.643
No	71 (67)	46 (65)	25 (71)	
Yes	35 (33)	25 (35)	10 (29)	
Anxiety, *n* (%)				0.679
No	71 (67)	49 (69)	22 (63)	
Yes	35 (33)	22 (31)	13 (37)	
Final temperature (°C), median (Q1–Q3)	75 (70–80)	77.5 (75–81.25)	70 (67.5–75)	<0.001
Operation time (min), *n* (%)				0.997
30	23 (22)	15 (21)	8 (23)	
35	33 (31)	22 (31)	11 (31)	
40	22 (21)	15 (21)	7 (20)	
45	10 (9)	7 (10)	3 (9)	
50	7 (7)	5 (7)	2 (6)	
55	4 (4)	2 (3)	2 (6)	
60	4 (4)	3 (4)	1 (3)	
70	3 (3)	2 (3)	1 (3)	

### Feature selection

**Figure 2:**
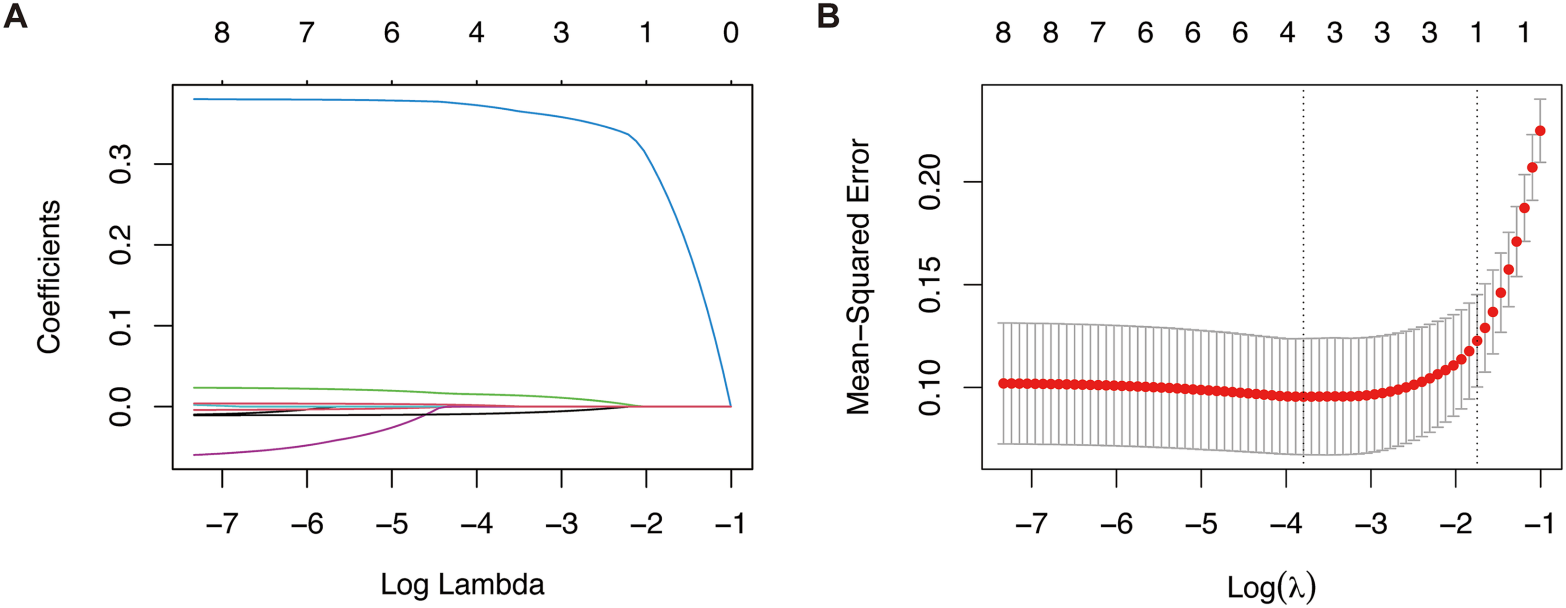
Presentation of the outcomes of the LASSO regression analysis. **(A)** LASSO regression model screening variable trajectories. **(B)** LASSO Regression Model Factor Selection.

Figure 2 shows the results of feature screening based on LASSO algorithm. Figure 2A illustrates the coefficient paths of the LASSO regression model, showing the trajectory of variable selection. The left dashed line indicates the lambda value corresponding to the minimum mean cross-validated error (lambda.min), while the right dashed line represents the most regularized model within one standard error of the minimum (lambda.1se) (Figure 2B). In this study, the optimal penalty parameter (*λ*) was determined using 10-fold cross-validation based on the minimum criterion (lambda.min, λ = 0.0225), which minimized the binomial deviance while maintaining a robust balance between model fit and parsimony. Based on this parameter, four features with non-zero coefficients—namely duration, JRS, final temperature, and operation time—were selected as the optimal predictors for subsequent analysis. We used the backward stepwise method to fit the multivariable logistic regression model. In multivariable logistic regression, three factors were associated with recurrence independently: ‘Duration’, ‘JRS’ and ‘Final temperature’ (Table 2). With 35 documented recurrence events and these 3 independent significant predictors retained in the final model, the true Events Per Variable (EPV) ratio fully satisfies the universally recommended threshold of EPV ≥ 10, thereby ensuring the robustness of the regression coefficients and minimizing the risk of model overfitting (25, 26). We further employed the VIF to assess multicollinearity among variables, thereby refining the selection process. Variables with VIF values exceeding 10 were indicative of severe multicollinearity, The VIF values for three variables are less than 5, indicating no multicollinearity exists (Supplementary Table 1).

**Table 2:** Multivariable logistic regression analysis.

Variable	β (Estimate)	SE	Wald χ^2^	OR	95% CI	*p* value
Duration	0.409	0.172	5.64	1.50	1.07–2.10	0.017
JRS	2.902	0.810	12.83	18.21	3.73–88.94	<0.001
Operation time	0.071	0.038	3.42	1.07	1.00–1.15	0.064
Final temperature	−0.207	0.089	5.37	0.81	0.68–0.97	0.020

### Construction of the predictive nomogram

**Figure 3:**
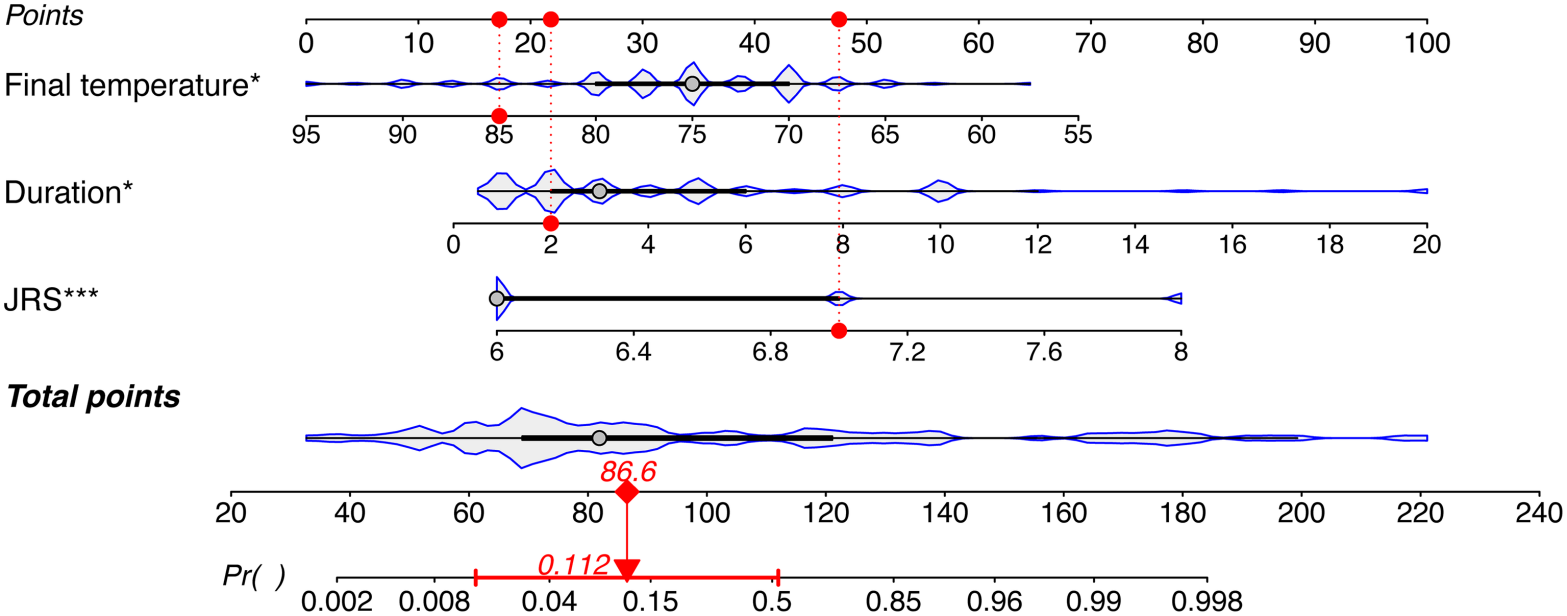
A nomogram for predicting recurrence was constructed based on the selected variables. The nomogram is applied through a two-step process. First, the value of each predictor is projected onto the “Points” axis at the top of the chart to obtain an individual score for each variable. Second, the individual scores are summed to yield a total score (potential range: 20–240), which corresponds to the predicted probability on the lower scale. Finally, a vertical line is drawn downward from the “Total Points” axis to estimate the probability of recurrence.

We included the variables selected from the multiple logistic regression analysis in the nomogram prediction model. The outcome indicator is whether patients with MS may have a recurrence 1 year after facial nerve radiofrequency ablation. We created the nomogram (Figure 3) and used the ruler above each risk factor on the nomogram to obtain the individual score for that factor. We added the scores for all risk factors to obtain the total score that can be used to estimate the probability of recurrence in the corresponding patient 1 year after facial nerve radiofrequency ablation. A higher total score indicates a greater likelihood of recurrence.

### Interpretation of the nomogram model

**Figure 4:**
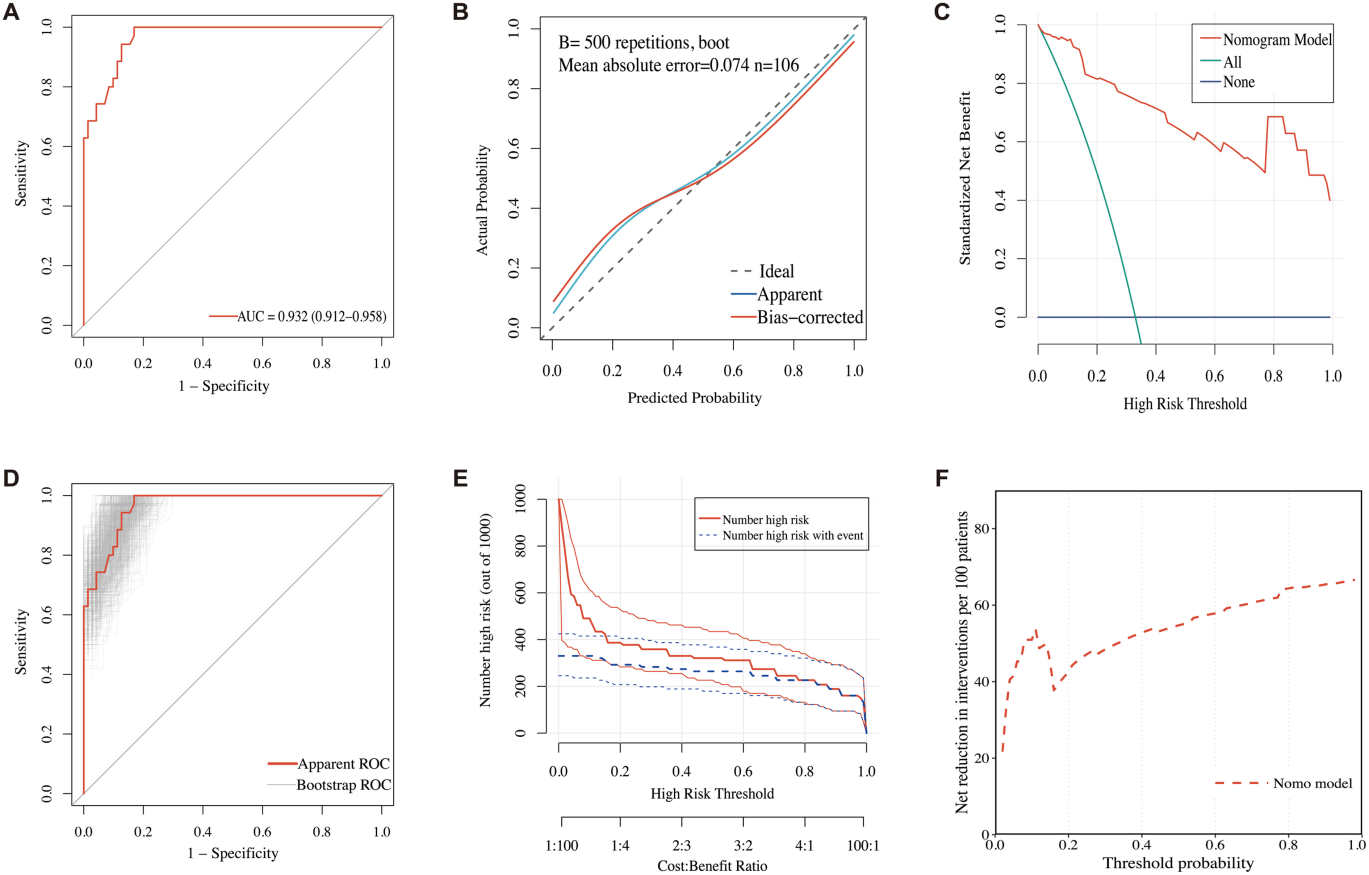
Performance evaluation of the logistic regression prediction model. **(A)** Receiver operating characteristic (ROC) curve. **(B)** Calibration plot comparing predicted and observed probabilities. **(C)** Decision curve analysis (DCA) evaluating clinical utility. **(D)** Bootstrap-validated ROC curve (500 replicates) with shaded area representing bootstrap AUC distribution and solid line indicating apparent performance. **(E)** Clinical impact curve showing population level effects. **(F)** Net intervention reduction analysis quantifying potential clinical benefits.

Receiver operating characteristic (ROC) curves were constructed to evaluate the predictive performance of the nomogram. As shown in Figure 4A, the area under the ROC curve (AUC) was 0.932 (95% CI: 0.912–0.958), indicating excellent discriminative ability. To assess model robustness, internal validation was performed using 500 bootstrap resamples (Figure 4D), yielding a bias-corrected mean AUC of 0.918 (95% CI: 0.898–0.962), which demonstrates good generalizability and consistency. Model calibration was further evaluated using bootstrap resampling (500 iterations). The calibration curve in Figure 4B shows close agreement between predicted and observed probabilities, indicating good calibration and predictive accuracy. Using the optimal cutoff determined by the Youden index (0.111), the model achieved a sensitivity of 1.000, specificity of 0.831, accuracy of 0.887, precision of 0.745, and an F1-score of 0.854. According to the decision curve analysis (DCA), the X-axis represents the threshold probability for predicting recurrence risk, and the Y-axis represents the net benefit. The red line represents the net benefit of decisions based on the nomogram model, the green line represents the strategy of treating all patients, and the blue line represents treating no patients (27). Notably, across a wide range of threshold probabilities (0.02–0.98), the nomogram model provides greater net benefit than both the “treat-all” and “treat-none” strategies. This performance indicates that the model maintains favorable clinical utility regardless of the risk threshold selected by clinicians (Figure 4C). Clinical impact curve (CIC) analysis demonstrated that when the threshold probability exceeded 0.6, there was a high degree of concordance between the predicted high-risk population and the observed high-risk population, confirming strong clinical relevance (Figure 4E). The net reduction in interventions curve indicates that the X-axis represents the threshold probability, and the Y-axis represents the number of unnecessary interventions avoided per 100 patients (Figure 4F). When the threshold probability ranged from 0.4 to 0.6, approximately 40 to 60 unnecessary interventions per 100 patients could be avoided, further supporting the clinical value of the model within this range. Based on the analysis above, the predictive model not only demonstrates high predictive accuracy but also has substantial clinical utility in supporting decision-making and optimizing healthcare resource allocation.

### Nomogram online web page development

**Figure 5:**
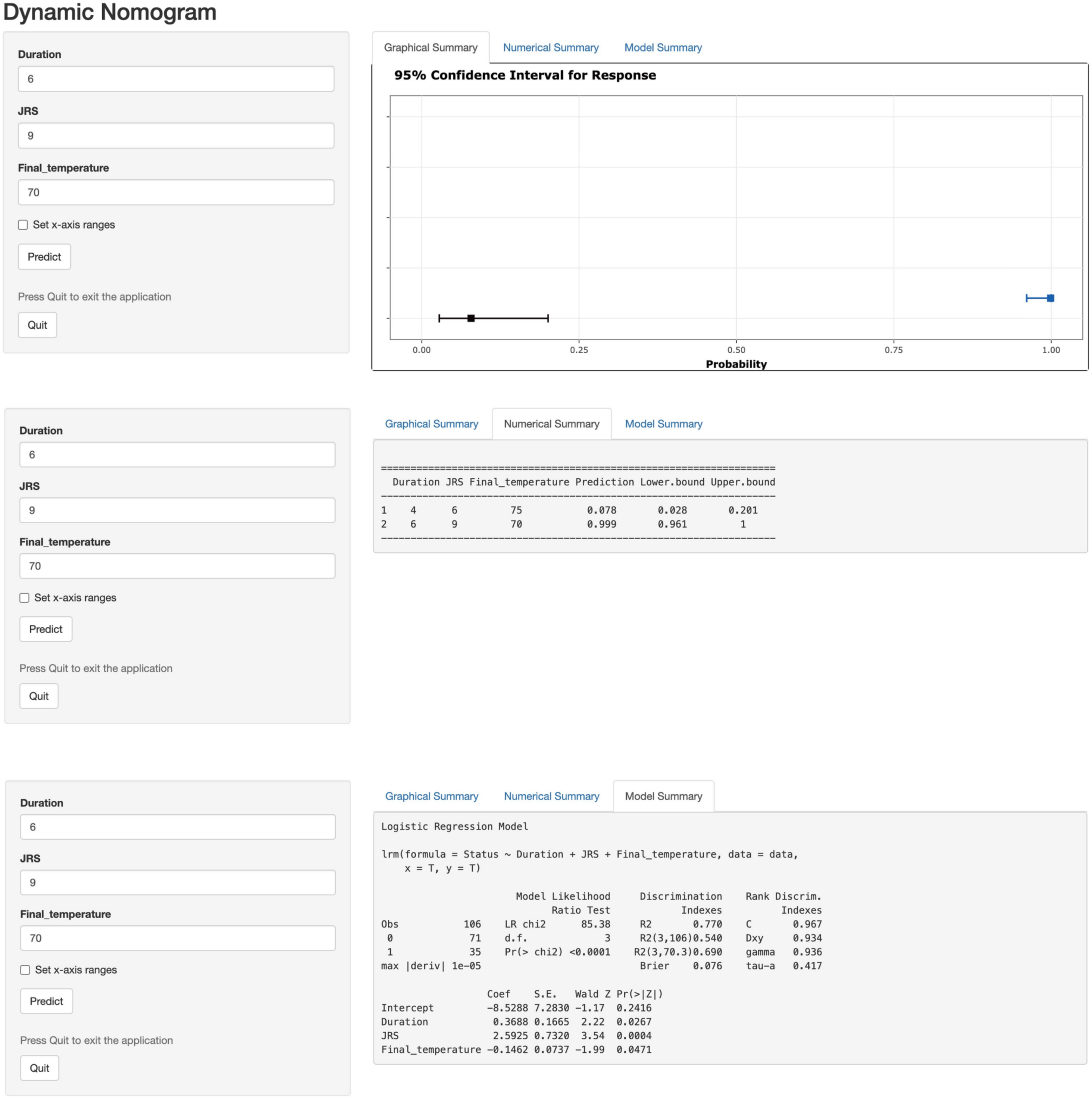
An online dynamic nomogram, accessible at https://jxsdyywz.shinyapps.io/dynnomapp/, is an interactive tool that allows clinicians to input individual patient data and obtain real-time predictions of recurrence risk after surgery.

To facilitate clinical use, we developed an online version of the model using Shiny (Figure 5), The model is available at: https://jxsdyywz.shinyapps.io/dynnomapp/. This tool allows clinicians to input patient-specific clinical data and instantly estimate the risk of postoperative recurrence, thereby supporting more accurate clinical decision-making. In addition to improving efficiency and accessibility, it also enhances personalized patient management.

## Discussion

In this retrospective study, we developed and internally validated a nomogram for predicting 1-year recurrence after CT-guided facial nerve radiofrequency ablation in patients with Meige syndrome presenting predominantly with blepharospasm. Three readily available clinical variables—preoperative JRS score, disease duration, and final radiofrequency temperature—were identified as independent predictors of recurrence. The resulting model demonstrated excellent discrimination, good calibration, and favorable clinical utility on decision curve analysis. To facilitate individualized risk assessment, we further developed a web-based dynamic nomogram for routine clinical application.

Although the pathophysiology of Meige syndrome remains incompletely understood, accumulating evidence suggests that abnormal basal ganglia-thalamocortical network activity, impaired inhibitory control, and maladaptive neuroplasticity contribute to sustained dystonic movements (5, 28). Current therapeutic strategies target different levels of this motor pathway. Botulinum toxin primarily blocks neuromuscular transmission (29, 30), whereas DBS modulates abnormal central motor circuits through GPi or STN stimulation (12, 31). In contrast, facial nerve radiofrequency ablation interrupts the peripheral transmission of pathological motor output without directly modifying the central generator. Consequently, recurrence remains inevitable in a subset of patients, highlighting the need for reliable individualized prediction tools to guide postoperative surveillance and personalized management.

First, the JRS score, as a quantitative measure of blepharospasm severity, reflects the intensity of abnormal motor activity (17). Higher JRS scores indicate more pronounced abnormal neuronal discharges and more widespread network dysfunction. Although radiofrequency ablation alleviates symptoms by interrupting peripheral nerve conduction, in patients with higher JRS scores, persistent and strong central pathological discharges may accelerate peripheral axonal regeneration or collateral reinnervation, thereby contributing to recurrence. Previous studies have similarly demonstrated that baseline symptom severity is closely associated with treatment outcomes (32). Therefore, patients with higher baseline JRS scores may require closer postoperative surveillance and individualized follow-up strategies.

Second, prolonged disease duration may reflect the long-standing presence of abnormal motor patterns within the central nervous system, leading to neuroplastic changes and functional reorganization in the basal ganglia–thalamocortical circuit (33, 34). Evidence suggests that patients with chronic dystonia exhibit reduced inhibitory control and increased cortical excitability, which may limit the long-term effectiveness of peripheral interventions (35). This finding is also consistent with previous studies on predictors of DBS outcomes, indicating that with longer disease duration, maladaptive neuroplasticity becomes progressively consolidated and less amenable to modulation (36, 37). These findings further support early intervention before maladaptive motor network reorganization becomes irreversible.

Finally, our multivariable analysis identified the final ablation temperature as an independent protective factor against 1-year recurrence. This association is biologically plausible because the durability of neural conduction block depends on the extent of thermal tissue injury (38). Lower ablation temperatures may produce relatively limited thermal lesions, resulting primarily in reversible myelin injury or low-grade axonal damage while preserving the endoneurial architecture and Schwann cell basal lamina. Under these conditions, axonal regeneration and collateral sprouting can occur more readily, facilitating restoration of pathological motor conduction and increasing the likelihood of symptom recurrence (39). In contrast, higher ablation temperatures generate more complete protein denaturation and coagulation necrosis, producing extensive axonal disruption and Wallerian degeneration, thereby interrupting abnormal motor signal transmission more durably (40). Experimental studies have demonstrated that lesion size and the extent of neural destruction increase in a temperature-dependent manner during radiofrequency thermocoagulation (41). Similarly, clinical studies have likewise shown that higher ablation temperatures are associated with improved long-term symptom control (42, 43), which is consistent with our observation that higher final temperatures were associated with lower risk of postoperative recurrence. Nevertheless, excessively high temperatures may enlarge the thermal field beyond the intended target at the stylomastoid foramen, significantly increasing the risk of severe facial nerve palsy. Consequently, the optimal therapeutic strategy should balance durable symptom control against neurological safety through precise, individualized temperature control rather than indiscriminate thermal escalation.

Nevertheless, several limitations should be acknowledged. First, this study was a single-center, retrospective analysis with a relatively limited sample size and a restricted number of recurrence events, which may have constrained statistical power and introduced inherent selection biases. Although internal validation via bootstrap resampling demonstrated robust performance, our model lacks external validation in an independent, geographically distinct cohort. Consequently, a potential risk of model overfitting cannot be entirely ruled out, which may compromise the immediate generalizability of our findings to other clinical settings. Future large-scale, prospective, multi-center studies are warranted to externally validate the transportability, calibration, and clinical utility of this nomogram. Second, intermediate postoperative assessments at 3 and 6 months were not consistently available and therefore could not be included in the analysis. Although recurrence at 1 year was selected as the primary endpoint because it represents a clinically meaningful measure of medium-term treatment durability, this follow-up duration remains insufficient to fully evaluate the long-term stability of radiofrequency ablation outcomes. Furthermore, while patients receiving major adjunct therapies (e.g., repeat ablation, DBS, or botulinum toxin) were strictly excluded or controlled, residual confounding from minor, unmeasured variations in post-procedural management cannot be completely ruled out. Future prospective trials with rigid, multi-point follow-up intervals and standardized post-operative care protocols are warranted to corroborate our findings. Third, the prediction model was developed primarily using routinely available clinical and procedural variables. Although these variables facilitate practical clinical application, biomarkers reflecting central neural network dysfunction, such as functional neuroimaging and electrophysiological indices, were not included. Integration of multimodal biomarkers may further improve both predictive performance and biological interpretability in future studies. Fourth, unlike previous epidemiological studies reporting a female predominance in Meige syndrome, our surgical cohort contained a higher proportion of male patients. This discrepancy is likely attributable to referral bias, selection bias associated with tertiary-center surgical treatment, and regional differences in healthcare utilization. Moreover, our model was developed exclusively in patients with Meige syndrome presenting predominantly with blepharospasm to maintain cohort homogeneity. Therefore, caution should be exercised when extrapolating the present prediction model to populations with different sex distributions or to other clinical subtypes of Meige syndrome. Finally, the accompanying dynamic nomogram is intended for individualized risk estimation within the range of patient characteristics represented in the development cohort, and its predictive performance should be interpreted cautiously when applied to substantially different populations. Overall, the present study provides a practical and interpretable prediction model for individualized recurrence risk assessment after facial nerve radiofrequency ablation in patients with Meige syndrome presenting predominantly with blepharospasm. Nevertheless, prospective multicenter studies with independent external validation are warranted to confirm its generalizability and clinical applicability before routine clinical implementation.

## Conclusion

This study identified disease duration, preoperative JRS score, and final temperature as independent predictors of 1-year recurrence following CT-guided facial nerve radiofrequency ablation in patients with MS. Based on these variables, a nomogram model was developed and demonstrated superior predictive performance and clinical utility, offering a reliable tool for individualized risk assessment and postoperative management.

## Data Availability

The raw data supporting the conclusions of this article will be made available by the authors, without undue reservation.
