# Supplementary material for: Development and validation of a nomogram for predicting recurrence in patients with Meige syndrome after radiofrequency ablation
Source: Front Neurol. 2026 Jul 14;17:1841516. doi: 10.3389/fneur.2026.1841516 (PMC13407107; doi:10.3389/fneur.2026.1841516)
Supplement: Supplementary file 1 [file Table_1.DOC]

**Supplementary Materials for**

**Development and validation of a nomogram for predicting recurrence in patients with Meige syndrome after radiofrequency ablation**

STROBE Statement—Checklist of items that should be included in reports of cohort studies

|  | Item No | Recommendation |  | Page No. |  |
| --- | --- | --- | --- | --- | --- |
| **Title and abstract** | 1 | (*a*) Indicate the study’s design with a commonly used term in the title or the abstract |  | Page 1, Lines 2–4; Page 1, Lines 20–22 |  |
|  |  | (*b*) Provide in the abstract an informative and balanced summary of what was done and what was found |  | Page 1–2, Lines 17–39 |  |
| Introduction | | |  |  |  |
| Background/rationale | 2 | Explain the scientific background and rationale for the investigation being reported |  | Page 2–3, Lines 46–70 |  |
| Objectives | 3 | State specific objectives, including any prespecified hypotheses |  | Page 3, Lines 72–74 |  |
| Methods | | |  |  |  |
| Study design | 4 | Present key elements of study design early in the paper |  | Page 3-4, Lines 77–82 |  |
| Setting | 5 | Describe the setting, locations, and relevant dates, including periods of recruitment, exposure, follow-up, and data collection |  | Page 4, Lines 79–82 |  |
| Participants | 6 | (*a*) Give the eligibility criteria, and the sources and methods of selection of participants. Describe methods of follow-up |  | Page 4, Lines 82–89 |  |
|  |  | (*b*) For matched studies, give matching criteria and number of exposed and unexposed |  | Not applicable. |  |
| Variables | 7 | Clearly define all outcomes, exposures, predictors, potential confounders, and effect modifiers. Give diagnostic criteria, if applicable |  | Page 5, Lines 127–128 |  |
| Data sources/ measurement | 8* | For each variable of interest, give sources of data and details of methods of assessment (measurement). Describe comparability of assessment methods if there is more than one group |  | Page 5, Lines 123–127 |  |
| Bias | 9 | Describe any efforts to address potential sources of bias |  | Page 4, Lines 89–95 & Page 11, Lines 280–282 |  |
| Study size | 10 | Explain how the study size was arrived at |  | Page 4, Lines 97–98 |  |
| Quantitative variables | 11 | Explain how quantitative variables were handled in the analyses. If applicable, describe which groupings were chosen and why |  | Page 5-6, Lines 130–135 |  |
| Statistical methods | 12 | (*a*) Describe all statistical methods, including those used to control for confounding |  | Page 5-6, Lines 130–142 |  |
|  |  | (*b*) Describe any methods used to examine subgroups and interactions |  | Not applicable. |  |
|  |  | (*c*) Explain how missing data were addressed |  | Page 4, Lines 91 |  |
|  |  | (*d*) If applicable, explain how loss to follow-up was addressed |  | Page 7, Lines 154-155 |  |
|  |  | (*e*) Describe any sensitivity analyses |  | Not applicable. |  |
| Results | | |  |  |  |
| Participants | 13* | (a) Report numbers of individuals at each stage of study—eg numbers potentially eligible, examined for eligibility, confirmed eligible, included in the study, completing follow-up, and analysed |  | Figure 1 |  |
|  |  | (b) Give reasons for non-participation at each stage |  | Figure 1 |  |
|  |  | (c) Consider use of a flow diagram |  | Figure 1 |  |
| Descriptive data | 14* | (a) Give characteristics of study participants (eg demographic, clinical, social) and information on exposures and potential confounders |  | Page 6, Lines 154–158 & Table 1 |  |
|  |  | (b) Indicate number of participants with missing data for each variable of interest |  | Page 6, Line 166 & Table 1 |  |
|  |  | (c) Summarise follow-up time (eg, average and total amount) |  | Page 5, Lines 127–128 |  |
| Outcome data | 15* | Report numbers of outcome events or summary measures over time |  | Page 7, Lines 157–158 |  |
| Main results | 16 | (*a*) Give unadjusted estimates and, if applicable, confounder-adjusted estimates and their precision (eg, 95% confidence interval). Make clear which confounders were adjusted for and why they were included |  | Page 7, Lines 158–174 & Table 2 |  |
|  |  | (*b*) Report category boundaries when continuous variables were categorized |  | Not applicable. |  |
|  |  | (*c*) If relevant, consider translating estimates of relative risk into absolute risk for a meaningful time period |  | Page 8, Lines 183–189& Figure 3 |  |
| Other analyses | 17 | Report other analyses done—eg analyses of subgroups and interactions, and sensitivity analyses |  | Page 8-9, Lines 191–217, Figure 4 |  |
| Discussion | | |  |  |  |
| Key results | 18 | Summarise key results with reference to study objectives |  | Page 9-10, Lines 225–232 |  |
| Limitations | 19 | Discuss limitations of the study, taking into account sources of potential bias or imprecision. Discuss both direction and magnitude of any potential bias |  | Page 11-12, Lines 281–312 |  |
| Interpretation | 20 | Give a cautious overall interpretation of results considering objectives, limitations, multiplicity of analyses, results from similar studies, and other relevant evidence |  | Page 10-11, Lines 233–280 |  |
| Generalisability | 21 | Discuss the generalisability (external validity) of the study results |  | Page 12, Lines 285–287& 304-306 |  |
| Other information | | |  |  |  |
| Funding | 22 | Give the source of funding and the role of the funders for the present study and, if applicable, for the original study on which the present article is based |  | Page 11, Lines 331–336 |  |

**Supplementary Table 1. Variance Inflation Factor**

| Variable | VIF |
| --- | --- |
| Duration | 1.029 |
| JRS | 1.028 |
| Final temperature | 1.003 |

Supplementary Table 2. Regression coefficients of the final prediction model

| Predictor | Coefficient (β) |
| --- | --- |
| Intercept | -8.5288 |
| Duration | 0.3688 |
| JRS | 2.5925 |
| Final Temperature | -0.1462 |

Supplementary Method S1. Final Logistic Regression Equation

The final multivariable logistic regression model used for nomogram
construction was:

LP = −8.5288 + 0.3688 × Duration + 2.5925 × JRS − 0.1462 × Final Temperature

Predicted recurrence probability was calculated as:

P = 1 / (1 + e⁻ᴸᴾ)
